# Systematic Review: Guideline-Based Approach for the Management of Asthma and Subtypes via Chinese Medicine

**DOI:** 10.1155/2021/4319657

**Published:** 2021-01-07

**Authors:** Lin Ho Wong, Louisa Tay, Robby Miguel W. J. Goh, Tai Joum Tan, Ruishu Zhou, Aaron Kwun Hang Ho, Pang Ong Wong

**Affiliations:** ^1^Ong Fujian Chinese Physician Hall, Singapore; ^2^Singapore College of Traditional Chinese Medicine, Singapore; ^3^University College London, London, UK; ^4^Ministry of Health Holdings, Singapore; ^5^Chang Chun University of Traditional Chinese Medicine, Changchun, China

## Abstract

**Background:**

Asthma is a chronic condition that results in the inflammation and narrowing of airways, often clinically presenting as wheeze and shortness of breath. Little is known of the mechanisms of action (MOA) of herbs used to treat asthma. The aim of this study is to review existing data regarding known MOA of traditional Chinese medicine which will aid in the understanding of possible interactions between Western drugs and Chinese herbs as well as the standardization of management via a proposed guideline to improve patient safety and possible synergism in the long term.

**Methods:**

We searched through 5 databases for commonly prescribed herbs and formulas for asthma and narrowed down the search to identify the underlying MOA of individual herbs that could specifically target asthma symptoms. We included studies that stated the MOA of individual herbs when used for treating symptoms of asthma, excluding them if they are described as part of a formula.

**Results:**

A total of 26 herbs commonly prescribed for asthma with known mechanism of action were identified. Herbs used for asthma were found to have similar MOA as that for drugs. Based on existing GINA guidelines, a guideline is proposed which includes a total of 5 steps depending on the severity of asthma and the herbs' MOA. 16 formulas were subsequently identified for the management of asthma, which consist of 12 “stand-alone” and 4 “add-on” formulas. “Stand-alone” formulas used independently for asthma generally follow the GINA guidelines but do not proceed beyond step 3. These formulas consist mainly of beta-agonist and steroid-like effects. “Add-on” formulas added as adjunct to “stand-alone” formulas, however, mainly act on T helper cells or have steroid-like effects.

**Conclusion:**

Through the understanding of MOA of herbs and their respective formulas, it will ensue greater patient safety and outcomes.

## 1. Introduction

There has been an increasing trend in the use of complementary and alternative medicine (CAM) [1] since its acknowledgement by the World Health Organization (WHO) in 2018 [2]. Herbs are being used by 75% of the people in the world for their basic healthcare needs [3]. Despite the dominance of Western evidence-based medicine in healthcare settings, people occasionally seek traditional Chinese medicine (TCM) treatments, a widely used form of CAM, for medical conditions such as chronic pain and allergies, including, but not limited to, asthma.

Asthma is a heterogeneous disease that is characterized by chronic, reversible airway inflammation that involves eosinophil granulocytes, mast cells, T cells, and an array of cytokines. As curative treatments for asthma are still not available, patients are increasingly seeking CAM for add-on treatments, resulting in growing concerns over the concomitant use of Western medicine and CAM herbs [4]. Furthermore, most CAM herbs used for asthma have unknown mechanism of actions (MOA). This could potentially lead to dangerously high concentration of constituents with the same MOA, which may lead to severe side effects.

While Western doctors typically manage asthma using the Global Initiative for Asthma (GINA) guidelines based on the disease severity, TCM adopts a less guideline-based and more individualized therapy via a holistic view of the patient's pathophysiology, resulting in a discrepancy in herbal prescribing between TCM physicians [5]. With increasing scientific evidence to support the use of TCM for asthma [6], it is crucial for both medical doctors and TCM physicians to be aware of the potential risks that come with the simultaneous use of prescribed drugs and herbal medicine.

This review aims to categorise commonly used herbs and empirical prescriptions of TCM based on known MOA of Western medicines to allow both Western and TCM physicians to have a better understanding of how drugs and herbs interact. This will hopefully lead to increased patient safety and possible synergism in the long run, giving patients more choices for an individually tailored treatment and better treatment outcomes.

## 2. Methods

### 2.1. Criteria for considering Studies for This Review

#### 2.1.1. Types of Studies

All studies eliciting the mechanism of action of a specific herb in the context of asthma, regardless of the model. Studies which involved the use of multiple herbs simultaneously (i.e., formulas) were excluded.

### 2.2. Search Methods for Identification of Studies

#### 2.2.1. Electronic Searches

For this review, we searched EMBASE, PubMed, CENTRAL, Wanfang, and China National Knowledge Infrastructure (CNKI).

### 2.3. Identifying the Herbs

**Figure 1:**
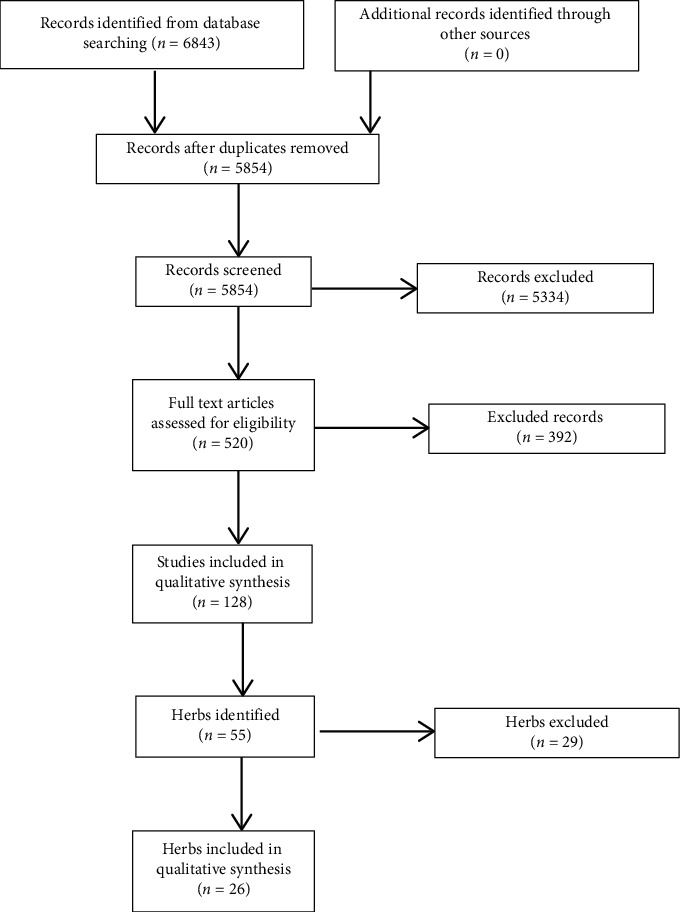
Study flow diagram of the identification of commonly used herbs for asthma.

We first conducted a search to identify herbs which are currently commonly prescribed by TCM physicians by identifying systematic reviews on frequently used herbs or formulas and extracted the relevant herbs from the formulas, using the terms “asthma AND TCM OR Traditional Chinese Medicine OR alternative medicine” in EMBASE, PubMed, CENTRAL, Wanfang, and CNKI (Figure 1). The list was subsequently confirmed with a senior TCM physician specialising in TCM Formula-logy.

**Figure 2:**
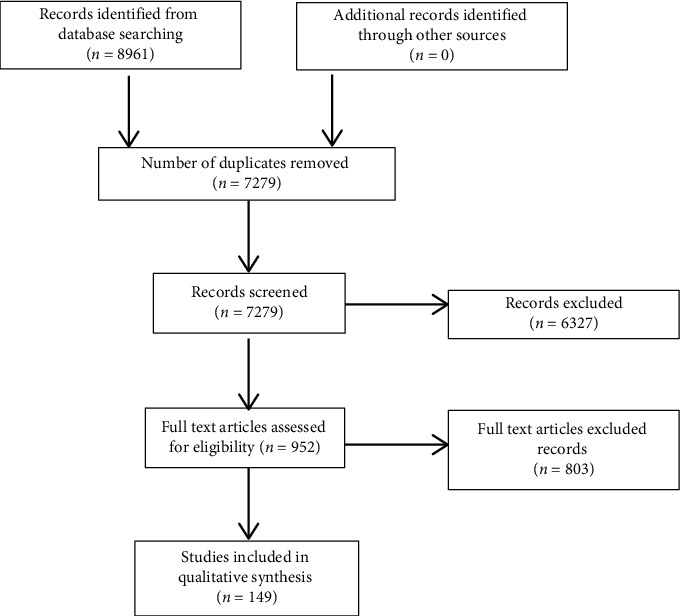
Study flow diagram for the identification of articles of commonly used herbs.

We then extracted the Latin and Mandarin names of the herbs used and identified those used primarily for the treatment of asthma, before searching electronic databases for their mechanisms of action (Figure 2). Herbs prescribed for supportive treatment were excluded.

### 2.4. EMBASE, PubMed, and Cochrane

#### 2.4.1. CNKI (Overseas CNKI) and Wanfang (Based on the Translations of the Keywords Used in English Medium)

1. “*Glycyrrhiza uralensis*” AND “asthma”
2. “*Prunus armeniaca*” AND “asthma”
3. “*Pinellia ternata*” AND “asthma”
4. “*Asarum sieboldii*” AND “asthma”
5. “*Pheretima aspergillum*” AND “asthma”
6. “*Aster tataricus*” AND “asthma”
7. “*Fritillaria cirrhosa*” AND “asthma”
8. “*Lepidium apetalum*” AND “asthma”
9. “Pericarpium Citri Reticulatae” AND “asthma”
10. “Cortex Mori” AND “asthma”
11. “*Ephedra sinica*” AND “asthma”
12. “*Zingiber officinale*” AND “asthma”
13. “*Tussilago farfara*” AND “asthma”
14. “*Platycodon grandifloras*” AND “asthma”
15. “*Fritillaria thunbergii*” AND “asthma”
16. “*Paeonia lactiflora*” AND “asthma”
17. “*Magnolia officinalis*” AND “asthma”
18. “*Bupleurum chinense*” AND “asthma”
19. “*Scutellaria baicalensis*” AND “asthma”
20. “*Perilla frutescens*” AND “asthma”
21. “Anemarrhena Rhizoma” AND “asthma”
22. “*Gypsum fibrosum*” AND “asthma”
23. “Eriobotryae Folium” AND “asthma”
24. “Cinnamomi Ramulus” AND “asthma”
25. “*Zingiberis rhizoma*” AND “asthma”
26. “Schisandra Fructus” AND “asthma”

We did not impose any language or publication restrictions.

### 2.5. Data Collection and Analysis

#### 2.5.1. Selection of Studies

Four review authors (TJ, L, RS, and RM) independently reviewed the titles, abstracts, and keywords of all records retrieved to determine the studies to be assessed. We retrieved full articles for further assessment if the information given suggested that the study:

1. Discussed asthma
2. And administered any of 26 Chinese herbs identified through the initial search

A fifth review author (LH) acted as arbiter and resolved any differences in opinion.

#### 2.5.2. Data Analysis

**Figure 3:**
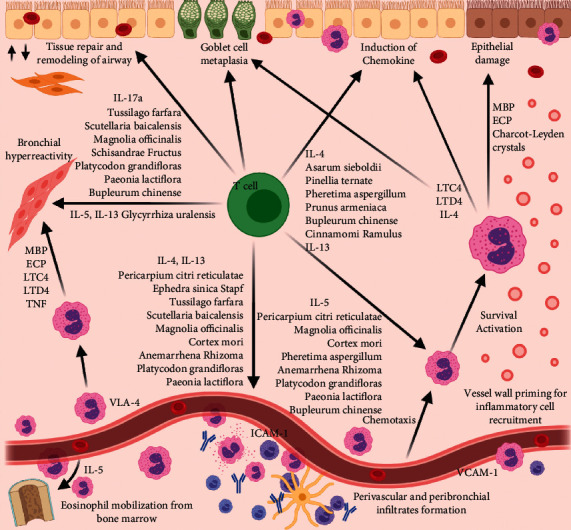
Chinese medicine targeted therapy on immunopathological pathways of asthma.

**Figure 4:**
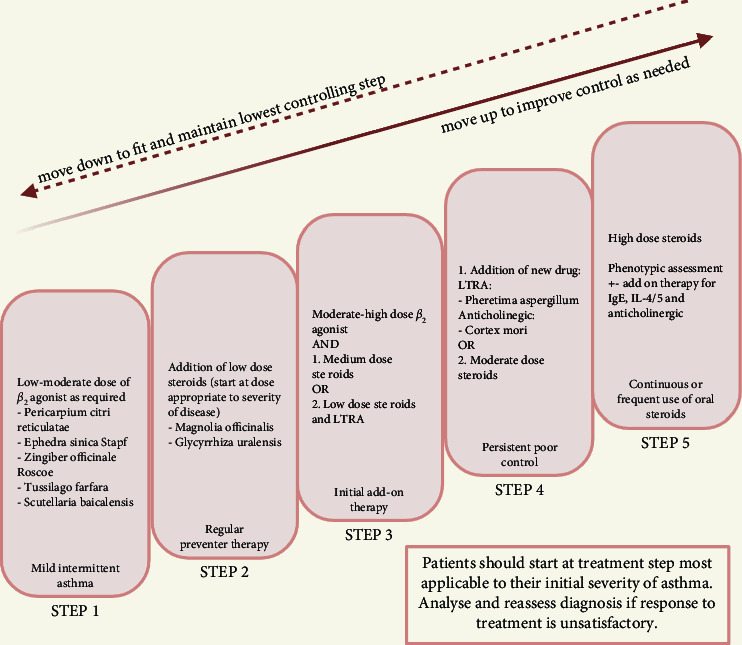
Proposed guideline for traditional Chinese medicine based on existing GINA.

We first identified the herbs relevant to treating asthma by the formulas which contain them, for reference purposes (Table 1). We then categorised the herbs according to their mechanisms of action (Table 2), plotted a schematic representation of the herbs on the asthma immunopathological pathway (Figure 3), and then compared them against GINA guidelines to identify clinical situations where herbs could see the potential use (Figure 4).

## 3. Results

### 3.1. Results of the Search to Identify Herbs of Interest

Our initial searches in online databases identified 6843 studies, of which 128 systematic reviews were relevant to our topic, yielding 55 herbs (Figure 1).

### 3.2. Included Herbs

Of the 55 herbs, 26 were used primarily for asthma treatment.

These included *Glycyrrhiza uralensis* [7–24], *Prunus armeniaca* [25, 26], *Pinellia ternata* [27], *Asarum sieboldii* [28, 29], *Pheretima aspergillum* [30–37], *Aster tataricus* [38, 39], *Fritillaria cirrhosa* [40–43], *Lepidium apetalum* [44], Pericarpium Citri Reticulatae [45–47], Cortex Mori [48–52], *Ephedra sinica* Stapf [53–56], *Zingiber officinale* Roscoe [57], *Tussilago farfara* [58–61], *Platycodon grandifloras* [62–65], *Fritillaria thunbergii* [66, 67], *Paeonia lactiflora* [68–71], *Magnolia officinalis* [72–77], *Bupleurum chinense* [78, 79], *Scutellaria baicalensis* [80–86], Anemarrhena Rhizoma [87, 88], *Gypsum fibrosum*, Eriobotryae Folium [89], Cinnamomi Ramulus [90], *Zingiberis rhizoma*, Schisandra Fructus [91–93], and *Perilla frutescens* [94].

### 3.3. Results of the Search for Herb Mechanisms of Action

#### 3.3.1. Included Studies

Searching online databases yielded 8961 studies, of which 149 met the inclusion criteria (Figure 2). No studies were found for the herbs *Gypsum fibrosum* and *Zingiberis rhizoma*.

#### 3.3.2. Mechanisms of Action

We first extracted the proposed mechanisms of action of the herbs (Table 1) and categorised them according to the known main mechanisms of action of Western medicines important in the treatment of asthma. Based on known mechanisms, the herbs were divided broadly into beta-adrenergic agonist, steroid-like, anticholinergics, PDE antagonist, leukotriene antagonist, and herbs with monoclonal effects or those affecting signalling pathways (Table 2). The schematic representation on how herbs act on the immunopathological pathways of asthma is depicted in Figure 3. The herbs act mainly on IL-4, IL-5, IL-13, and IL-17A and T cells on asthmatic pathways, which act to mobilize inflammatory cells, tissue repair, and remodelling, causing bronchial hyperreactivity and induction of chemokines.

### 3.4. Proposed Guidelines in Relation to GINA Guidelines

The proposed guidelines (Figure 4), based on existing GINA guidelines, describe a stepwise approach to treating asthma, where increments in medication are made if existing treatment is insufficient for controlling a patient's symptoms [95].

  Step 1 involves using a low to moderate dose of beta-2-adrenoreceptor agonist as required. Herbs which work similarly include *Aster tataricus*, Pericarpium Citri Reticulatae, *Ephedra sinica* Stapf, *Zingiber officinale* Roscoe, *Tussilago farfara*, and *Scutellaria baicalensis*.
  Step 2 involves the addition of a low dose of corticosteroids. This can be achieved by adding *Magnolia officinalis* to the existing herbs or possibly by substituting them with *Glycyrrhiza uralensis* alone.
  Step 3 involves increasing the dose of the beta-2-adrenoreceptor agonist to a moderate-high dose and either using a medium dose of corticosteroid or adding a leukotriene receptor antagonist (LTRA) to the low-dose corticosteroid. *Pheretima aspergillum* is an LTRA and could potentially be useful.
  Step 4 involves the addition of either an anticholinergic or an LTRA or increasing to a moderate dose of corticosteroid, whichever was not undertaken during step 3. Cortex Mori has anticholinergic effects and could have potential use (Figure 4).
  Step 5 involves switching to a high dose of corticosteroid and the addition of drugs with effects such as inhibiting IgE, IL-4, or IL-5, guided by the phenotypic assessment by an asthma specialist. Herbs that may be useful for these purposes include *Asarum sieboldii*, *Pinellia ternata*, and *Prunus armeniaca* which inhibit IL-4, Schisandra Fructus which inhibits IL-17, *Lepidium apetalum* and *Perilla frutescens* which regulate Th2 helper cell activity, or Anemarrhena Rhizoma, Eriobotryae Folium, *Platycodon grandifloras*, *Paeonia lactiflora*, *Bupleurum chinense*, and Cinnamomi Ramulus which have a range of effects against multiple targets (Table 2).

### 3.5. Frequently Prescribed TCM Formulas in Asthma

A total of 16 formulas were identified as frequently prescribed for asthma (Table 3), of which 12 were “stand-alone formulas” which are usually prescribed independently and 4 were “add-on formulas” which are used in addition to “stand-alone formulas” during an acute exacerbation [96]. The MOA of the herbs identified in the formulas was analysed, which demonstrated that “stand-alone formulas” contain herbs with beta-agonist, steroid-like effects or leukotriene antagonist mechanisms, or a combination of beta-agonist and steroid-like effects/leukotriene antagonist. However, “add-on formulas” only had either steroid-like effects or regulated T helper cells.

## 4. Discussion

### 4.1. Mechanism of Action of Herbs and Concomitant Use of Drugs and Herbs

Despite theoretical differences between TCM and Western medicine, research is increasingly suggesting that herbal medicines have similar MOA to that of Western medicines (Table 1). The understanding of MOA allows characterisation of herbs in relation to Western medicines and improves prescription methods of TCM physicians. For example, TCM physicians will be able to select herbs with beta-agonist effects in patients presenting with acute asthma. It also allows minimisation of possible interactions between herbs and drugs if taken concurrently, and the use of readily available antidotes should occur. Recent research showed that up to 33.6% of patients in the United Kingdom consume both herbal and prescription medicine together [97]. This could result in overdose or interactions when herbs and drugs with similar MOA be consumed concurrently [98, 99]. Herbs such as *Gypsum fibrosum* and *Zingiberis rhizoma* which have no known MOA should not be used concurrently with Western medicines to prevent the occurrence of any unknown side effects.

### 4.2. Standardization of TCM Management of Asthma

TCM physicians traditionally adopt a “Syndrome-Based” approach in diagnosing patients which is determined by the symptoms of the patient, looks of the tongue, and character of the pulse [100]. “Syndrome-Based” approach means that patients with the same diagnosis given by Western doctors might be diagnosed as different syndromes by TCM physicians, and as such, different management would be given. It is also a relatively subjective approach depending on the consulting physician(s) [101]. It would likely result in differences in outcomes for patients. Through the understanding of the underlying MOA of each herb used for asthma, a proposed guideline (Figure 4) can thus be formulated based on existing GINA guidelines [95]. This would ensure a standardized uniform outcome for patients regardless of “syndromes” characterized by TCM physicians and prevent herbs with repetitive or unknown MOA from being prescribed.

Unlike drugs, herbs are usually consumed orally, and their duration of effect has not been elicited. The proposed guideline takes into account the differences in the mode of administration and durations of the effect of herbs despite the similarity in MOA. Similar to existing GINA guidelines, prescription will be stepwise and incremental according to the severity of their asthma, from step 1 to 5. Worsening of symptoms warrants moving up the ladder, and dose reductions can be made if symptoms improve. This will ensure adequate herbs with appropriate MOA and dosage are prescribed.

### 4.3. Phenotype Specific Asthma Therapeutic Targeting of Herbs

In addition to the standard therapeutic MOA for asthma, herbs also seem to regulate the activity of inflammatory cells and cytokines by acting on particular pathways in the immunopathology of asthma (Figure 3). This aids not only in understanding of the way herbs work but can also be used to target a particular phenotype of asthma, e.g., downregulation of IgE in allergen-induced asthma. Biologics such as omalizumab, which targets specific cytokines, have long been employed in the management of asthma. Herbs with similar effects could potentially be employed in a similar fashion as biologics.

The therapeutic interventions for different phenotypes include IL-17 antagonist (neutrophilic asthma), IL-4/13 antagonist and anti-IgE (allergen-induced asthma), corticosteroids and IL-5 antagonist (idiopathic eosinophilic asthma), and leukotriene antagonist (aspirin exacerbated asthma) [102].

Herbs which target certain phenotypes through their effect on cytokines (Table 2) should be added after standardized therapy has been given and phenotypic assessment has been conducted, in line with GINA guidelines. This serves as an individualised management for patients with different asthmatic phenotypes and will lead to improved treatment outcomes.

### 4.4. Analysis of TCM Formulas Used for Asthma

Herbs are typically prescribed in various combinations as “TCM formulas,” rather than individually. They can be broadly divided into “Stand-alone formulas” which can be used independently for diseases and “Add-on Formulas” which are usually added as adjuvants to “Stand-alone formulas” to enhance their therapeutic effects (Table 3). Interestingly, the analysis of “Stand-alone Formulas” has shown that despite the differences in herbs used, formulas generally consist of herbs with *β*-agonist and steroid-like effects as recommended by GINA guidelines [95]. On the other hand, “Add-on Formulas” consist of herbs with steroid-like effects or assist in the regulation of T helper cells. However, none of the formulas have proceeded beyond step 3 of the GINA guidelines which could involve the addition of anticholinergics or leukotriene antagonists.

In addition, some formulas consist of several herbs with the same MOA, e.g., Ma Xing Shi Gan Tang which comprises of *Ephedra sinica* Stapf and *Glycyrrhiza uralensis*, both of which exerts the *β*-agonistic effect and Gui Zhi Jia Hou Po Xing Ren Tang which is made up of *Magnolia officinalis* and *Glycyrrhiza uralensis*, both of which exert steroid-like effects. Unknowingly, Ma Xing Shi Gan Tang could result in arrhythmias as a result of excessive *β*-adrenergic activity, while Gui Zhi Jia Hou Po Xing Ren Tang could result in iatrogenic-induced Cushing syndrome, one of the well documented side effects of steroids especially in systemic steroids administration [103]. Gui Zhi Jia Hou Po Xing Ren Tang should therefore be avoided and let alone as an “Add-on Formula;” as the “Stand-alone Formula,” it is added onto and might already consist of steroidal elements. In addition, one of the existing formulas (Dong Shi Zhi Chuan Ji Ben Fang) also proceeded to include herbs with leukotriene antagonist activity without first including herbs with steroid-like effects as per GINA guidelines. This will likely result in the suboptimal management of patients with asthma.

## 5. Conclusion

With a rising number of patients seeking TCM [104], it is paramount for TCM physicians to understand the underlying MOA of herbs. This will allow the standardization of prescribing and result in a more guideline-based approach. In line with this approach, herbs with repetitive or unknown MOA should be avoided to reduce the risk of side effects. Both TCM physicians and doctors should also be wary of possible herb-drug interactions. A guideline-based approach will also allow greater continuity of care should a patient wish to transfer between TCM and Western medicine and prevent under or overmedication. However, patients should still seek the advice of their doctors prior to stopping medications.

Further research on *Gypsum fibrosum* and *Zingiberis rhizoma* to identify their underlying MOA is recommended.

## Figures and Tables

**Table 1:** Summary of MOA of herbs frequently used in asthma.

Herb species	Mechanism of action
*Glycyrrhiza uralensis*	*In murine models*
	(1) Bronchodilation
	LPS-induced NO production
	Attenuates acetylcholine- and carbachol-induced contractions
	(2) Anti-inflammatory
	Inhibit T lymphocytes, eosinophils, IgE, IL-13, and TNF-a
	Upregulate caspase-3 and Bax
	Steroid-like activities
	Downregulate Bcl-2
	(3) Mucolytic
	Inhibit MUC5AC gene expression, production and secretion via regulation of NF-*κ*B, STAT6, and HDAC2
	*In guinea pig models*
	(1) Bronchodilation
	Activate c-GMP and open calcium channels
	*Human airway epithelial cells*
	(1) Mucolytic
	Inhibit PMA-induced MUC5AC mucin production
	(2) Anti-inflammatory
	Inhibit Th2-associated cytokines
	*Human peripheral blood mononuclear cells*
	(1) Anti-inflammatory
	Phytohaemagglutinin-induced proliferation and inhibition of TNF-alpha, IFN-gamma, and IL-10 production
	*In vitro*
	(1) Anti-inflammatory
	Inhibit memory Th2 responses
	Inhibit IL-4 and Eotaxin-1 secretion

*Prunus armeniaca*	*In murine models*
	(1) Anti-inflammatory
	Reduce recruitment of eosinophils, macrophages, and lymphocytes
	Inhibit MAPK signalling and IL-4 activation
	Activate IFN-y

*Pinellia ternata*	*In murine models*
	(1) Anti-inflammatory
	Reduce recruitment of eosinophils
	Reduce IL-4 activation and IFN-y activation

*Asarum sieboldii*	*In guinea pig models*
	(1) Anti-inflammatory
	Inhibit IL-4 activation and histamine release
	(2) Signalling pathway regulation
	Regulate MMP-9 and TIMP-1 signalling

*Pheretima aspergillum*	*In murine models*
	(1) Anti-inflammatory
	Inhibit IL-4, IL-5, IgE, and TNF-*α* activation
	Inhibit eosinophil activation
	Regulate Th1/Th2 balance
	Inhibit NF-kB activation
	Inhibit production of NO, PGE2, TNF-a, iNOS, and COX-2
	Inhibit release of IL-1B and IL-6
	(2) Mucolytic
	Decrease collagen deposition
	Decrease mucin glycogen expression
	*In guinea pig models*
	(1) Anti-inflammatory
	Regulate IFN-y, IL-4, and LTB4 production
	*In human models*
	(1) Anti-inflammatory
	Inhibit TGF-B1 and SMAD2

*Aster tataricus* L. f.	*In vitro*
	(1) Anti-inflammatory
	Suppress NO production
	Inhibit production of PGE2, IL-6, IL-1B
	Inhibit expression of iNOS and COX-2 by inhibition of NF-KB activation
	(2) Signalling blockade
	Prevent activation of MAPK signalling cascade via inhibition of phosphorylation of c-Jun N-terminal kinases, extracellular signal-regulated kinases, and p38
	(3) Bronchodilation
	Activate of B2-adrenoceptors

*Fritillaria cirrhosa* D. Don	*In murine models*
	(1) Anti-inflammatory
	Suppress TH2 cytokines (IL-4, IL-5, and IL-13)
	Suppress IgE, histamine production
	Reduce eosinophilic accumulation
	Increase IFN-*γ* production
	(2) Block signalling pathways
	Inhibit ERK/MAPK signalling activation
	(3) Downregulate NOTCH 2 expression
	(4) Inhibit MMP-2, MMP-9, and TIMP-1

*Lepidium apetalum*	*In murine models*
	(1) Anti-inflammatory
	Reduce expression of type 2 cytokines
	Inhibit differentiation and activation of Th2 cytokines

Pericarpium Citri Reticulatae	*In murine models*
	(1) Anti-inflammatory
	Suppress eosinophil production
	*In guinea pig models*
	(1) Anti-inflammatory
	Downregulate expression of eosinophils and serum IgE, IL-4, and IL-5 levels
	(2) Bronchodilation
	Activation of B2-adrenoceptors

Cortex Mori	*In murine models*
	(1) Anti-inflammatory
	Enhancement of CD4(+)CD25(+)Foxp3(+) regulatory T cells and inhibition of Th2 cytokines such as interleukin (IL)-4, -5 and -13
	(2) Anticholinergic

*Ephedra sinica* Stapf	*In murine models*
	(1)Anti-inflammatory
	Reduce infiltration of inflammatory cells in the lung
	Regulate levels of inflammatory factors such as OVA-IgE, IL-4, and IL-13 and downregulate the expression of p65 NF-k B protein
	(2) Bronchodilator
	Activate a-, B1-, and B2-adrenoceptors

*Zingiber officinale* Roscoe	*In murine models*
	(1) Anti-inflammatory
	Inhibit Th2-mediated immune response
	(2) Bronchodilation
	Reduce Ca2+ influx in smooth muscle and promote *β*agonist-induced relaxation in human airway smooth muscle by suppressing phosphodiesterase 4D

*Tussilago farfara*	*In murine models*
	(1) Anti-inflammatory
	Regulate IgE, IL-4, and IL-13 levels
	Downregulate the expression of p65 NF-kB protein
	Inhibit NO, MAPKs, and NF-kB
	Suppress expression of PGE2, TNF-*α*, and HMGB1
	Reduce production of IL-4, IL-5, IL-13, and IL-17
	Reduce IgE in serum by regulating Th1/Th2 cells
	Increase HO-1 levels affecting Nrf2/HO-1 pathway
	(2) Mucolytic
	Decrease mucin production by regulating NF-kB

*Platycodon grandifloras*	*In murine models*
	Serum concentrations of NF-*κ*B, MMP-9, and TIMP-1 decreased significantly
	*In guinea pig models*
	(1) Anti-inflammatory
	Promote and regulate release of LXA4
	Reduce oxygen-free radicals
	Promote secretion of IFN-*γ*
	Regulate Th1/Th2 balance

*Fritillaria thunbergii*	*In guinea pig models*
	Inhibit PDE and prevent inactivation of cAMP

*Paeonia lactiflora*	*In murine models*
	(1) Anti-inflammatory
	Inhibit IL-22 and IL-13

*Magnolia officinalis*	*In murine models*
	(1) Anti-inflammatory
	Inhibit IL-4, IL-6, and IL-17
	Decrease serum MDA level
	Increase SOD and GSH-Px/p-JNK, NF-*κ*B, caspase-3, and *γ*H2 Ax levels
	Inhibition of the PI3 K/Akt signalling pathway by TLR2 and TLR4 receptors-Steroid-like activities

*Bupleurum chinense*	*In guinea pig models*
	(1) Anti-inflammatory
	Reduce eosinophil levels
	Reduce serum levels of IL-5 and TNF-*α*

*Scutellaria baicalensis*	*In murine models*
	(1) Anti-inflammatory
	Inhibit TGF-*β*1 and *α*-SMA, and decrease p-ERK1/2
	Inhibit phosphorylated p38 protein
	Inhibit IgE, IL-4, IL-5, IL-6, IL-17A
	Reduce STAT3 protein level
	Promote expression of FOXP3 protein
	Increase serum MDA levels
	Promote expression of FOXP3 protein
	Inhibit HMGB1
	Inhibit protein expression of *α*-SMA and TLR4
	Expression of GATA-3 and STAT6
	Suppress Th2 response
	Increase IL-10 levels

*Perilla frutescens*	None found

Anemarrhena Rhizoma	*In guinea pig models*
	Reduce serum NO, BALF, and ET-1

*Gypsum fibrosum*	None found

Eriobotryae Folium	*In murine models*
	Reduce CD4+
	Increase CD 8+
	Regulate CD4+/CD8+ dysfunction

Cinnamomi Ramulus	*In murine models*
	(1) Anti-inflammatory
	Inhibition of eosinophils, IFN-*γ*, IL-4, IgE, histamine, and *β*-hexosaminidase release

*Zingiberis rhizoma*	None found

Schisandra Fructus	*In murine models*
	(1) Anti-inflammatory
	Reduce EOS
	Increase SOD in serum
	Reduce MDA
	Inhibit TNF-*α*, IL-1*β,* and IL-6 expression
	Regulate the HMGB1/TLR4/NF-*κ*B signalling pathway

**Table 2:** Phenotype specific asthma therapeutic targeting of herbs.

Chinese name	Latin name	MOA targeting asthmatic subgroups	Targeted asthmatic phenotype
*β*-Adrenergic agonist			
Zi Wan	*Aster tataricus* L. f.	N/A	N/A
Chen Pi	Pericarpium Citri Reticulatae	(1) Inhibits IL-5 and reduction of eosinophil (2) Inhibits IgE and IL-4	(1) Idiopathic eosinophilic asthma (2) Allergen exacerbated asthma
Ma Huang	*Ephedra sinica* Stapf	Inhibits IL-4 and IL-13	Allergen exacerbated asthma
Sheng Jiang	*Zingiber officinale* Roscoe	N/A	N/A
Kuan Dong Hua	*Tussilago farfara*	(1) Inhibits IL-4, IL-13, and IgE (2) Inhibits IL-5 (3) Inhibits IL-17	(1) Allergen exacerbated asthma (2) Idiopathic eosinophilic asthma (3) Neutrophilic asthma
Huang Qin	*Scutellaria baicalensis*	(1) Inhibits IL-4, IL-13, and IgE (2) Inhibits IL-5 (3) Inhibits IL-17A	(1) Allergen exacerbated asthma (2) Idiopathic eosinophilic asthma (3) Neutrophilic asthma
Steroidal effects			
Hou Po	*Magnolia officinalis*	(1) Inhibits IL-4 and IL-13 (2) Inhibits IL-5 (3) Inhibits IL-17A (4) Inhibits leukotriene release	(1) Allergen exacerbated asthma (2) Idiopathic eosinophilic asthma (3) Neutrophilic asthma (4) Aspirin induced asthma
PDE inhibitor			
Zhe Bei	*Fritillaria thunbergii*	N/A	N/A
*β*-Adrenergic and steroidal effects			
Gan Cao	*Glycyrrhiza uralensis*	(1) Inhibits IgE and IL-13 (2) Steroidal effects and inhibits IL-5 antagonist (3) Steroidal effects	(1) Allergen exacerbated asthma (2) Idiopathic eosinophilic asthma (3) Neutrophilic asthma
Anticholinergic			
Sang Bai Pi	Cortex Mori	(1) Inhibits IL-4 and IL-13 (2) Inhibits IL-5	(1) Allergen exacerbated asthma (2) Idiopathic eosinophilic asthma
Leukotriene antagonist			
Di Long	*Pheretima aspergillum*	(1) Leukotriene antagonist (2) Inhibits IgE and IL-4 (3) Reduces eosinophils and inhibits IL-5	(1) Aspirin induced asthma (2) Allergen exacerbated asthma (3) Idiopathic eosinophilic asthma
Inhibition of IL-4			
Xi Xin	*Asarum sieboldii*	Inhibits IL-4 and histamine release	Allergen exacerbated asthma
Ban Xia	*Pinellia ternata*	Inhibits IL-4	Allergen exacerbated asthma
Ku Xing Ren	*Prunus armeniaca*	Inhibits IL-4	Allergen exacerbated asthma
Inhibition of IL-17			
Wu Wei Zi	Schisandra Fructus	Inhibits IL-17	Neutrophilic asthma
Regulation of T helper cells			
Ting Li Zi	*Lepidium apetalum*	Reduces the expression of Th2 cytokines and inhibits differentiation and activation of Th2 cells.	Allergen exacerbated asthma
Su Zi	*Perilla frutescens*	Suppression of allergen-specific Th2 response	Allergen exacerbated asthma
Pi Pa Ye	Eriobotryae Folium	Reduction of CD4+, rises CD 8+, and alters CD4+/CD8+ dysfunction	Allergen exacerbated asthma
Multiple monoclonal effects for asthma			
Zhi Mu	Anemarrhena Rhizoma	(1) Inhibits IL-5 and reduction of eosinophil (2) Inhibits histamine release, IgE, IL-4, and IL-13	(1) Idiopathic eosinophilic asthma (2) Allergen exacerbated asthma
Jie Geng	*Platycodon grandifloras*	(1) Inhibits IL-4 and IL-13 (2) Inhibits IL-5 (3) Inhibits IL-17	(1) Allergen exacerbated asthma (2) Idiopathic eosinophilic asthma (3) Neutrophilic asthma
Shao Yao	*Paeonia lactiflora*	(1) Inhibits IL-4 and IL-13 (2) Inhibits IL-5 and reduces eosinophil (3) Inhibits IL-17 and reduces neutrophil count	(1) Allergen exacerbated asthma (2) Idiopathic eosinophilic asthma (3) Neutrophilic asthma
Chai Hu	*Bupleurum chinense*	(1) Inhibits IL-4 and IgE (2) Inhibits IL-5 (3) Inhibits IL-17A	(1) Allergen exacerbated asthma (2) Idiopathic eosinophilic asthma (3) Neutrophilic asthma
Gui Zi	Cinnamomi Ramulus	(1) Inhibits IgE and IL-4	Allergen exacerbated asthma
Alteration of genetic expression/signaling pathway			
Bei Mu	*Fritillaria cirrhosa* D.Don	NA	NA

**Table 3:** Analysis of MOA of commonly used TCM formulas.

Formula (Chinese)	Formulas	Key ingredients	Main MOA	Individual/Add-on formula
三子养亲汤	San Zi Yang Qin Tang	*Perilla frutescens*	Regulation of Th2 cell	Add-on
三子汤	San Zi Tang	*Lepidium apetalum* and *Perilla frutescens*	Regulation of Th2 cell	Add-on
半夏厚朴汤	Ban Xia Hou Po Tang	*Pinellia ternata* and *Magnolia officinalis*	Steroid-like effects	Add-on
桂枝加厚朴杏仁汤	Gui Zhi Jia Hou Po Xing Ren Tang	Cinnamomi Ramulus, *Magnolia officinalis*, *Prunus armeniaca*, and *Glycyrrhiza uralensis*	Steroid-like effects	Add-on
射干麻黄汤	She Gan Ma Huang Tang	*Ephedra sinica* Stapf, *Aster tataricus* L. f., *Pinellia ternata*, Schisandra Fructus, *Asarum sieboldii*, and *Tussilago farfara*	*β*-Agonist	Individual
康连智方	Kang Lian Zhi Fang	*Ephedra sinica* Stapf, *Pinellia ternata*, *Asarum sieboldii*, and Schisandra fructus	*β*-Agonist	Individual
董氏治喘基本方	Dong Shi Zhi Chuan Ji Ben Fang	*Ephedra sinica* Stapf, *Prunus armeniaca*, and *Pheretima aspergillum*	*β*-Agonist and leukotriene antagonist	Individual
麻杏石甘汤	Ma Xing Shi Gan Tang	*Ephedra sinica* Stapf, *Glycyrrhiza uralensis*, and *Prunus armeniaca*	*β*-Agonist and steroid-like effects	Individual
三拗汤	San Ao Tang	*Ephedra sinica* Stapf, *Glycyrrhiza uralensis*, and *Prunus armeniaca*	*β*-Agonist and steroid-like effects	Individual
茯苓杏仁甘草汤	Fu Ling Xing Ren Gan Cao Tang	*Prunus armeniaca* and *Glycyrrhiza uralensis*	*β*-Agonist and steroid-like effects	Individual
六君子汤	Liu Jun Zi Tang	*Glycyrrhiza uralensis*, *Pinellia ternata*, and Pericarpium Citri Reticulatae	*β*-Agonist and steroid-like effects	Individual
治喘方	Zhi Chuan Fang	*Ephedra sinica* Stapf, *Asarum sieboldii*, *Glycyrrhiza uralensis*, Schisandra Fructus, and *Pinellia ternata*	*β*-Agonist and steroid-like effects	Individual
苓桂术甘汤	Ling Gui Shu Gan Tang	Cinnamomi Ramulus and *Glycyrrhiza uralensis*	*β*-Agonist and steroid-like effects	Individual
小柴胡汤	Xiao Chai Hu Tang	*Bupleurum chinense*, *Scutellaria baicalensis*, *Pinellia ternata*, and *Glycyrrhiza uralensis*	*β*-Agonist and steroid-like effects	Individual
小青龙汤	Xiao Qing Long Tang	*Ephedra sinica* Stapf, *Paeonia lactiflora*, *Asarum sieboldii*, *Glycyrrhiza uralensis*, Cinnamomi Ramulus, Schisandra Fructus, and *Pinellia ternata*	*β*-Agonist and steroid-like effects	Individual
芍药甘草汤	Shao Yao Gan Cao Tang	*Paeonia lactiflora* and *Glycyrrhiza uralensis*	*β*-Agonist and steroid-like effects	Individual

## Data Availability

The data used to support the findings of this study are available online.

## References

[1] Hori S., Mihaylov I., Vasconcelos J. C. (2008). Patterns of complementary and alternative medicine use amongst outpatients in Tokyo, Japan. *BMC Complementary and Alternative Medicine*.

[2] World Health Organization (2019). *WHO Global Report on Traditional and Complementary Medicine 2019*.

[3] Lin M.-H., Chang H.-T., Tu C.-Y., Chen T.-J., Hwang S.-J. (2015). Prevalence of polyherbacy in ambulatory visits to traditional Chinese medicine clinics in Taiwan. *International Journal of Environmental Research and Public Health*.

[4] Izzo A. A. (2012). Interactions between herbs and conventional drugs: overview of the clinical data. *Medical Principles and Practice*.

[5] Yan L., Rui L., Zibo O. (2015). Herb network analysis for a famous TCM doctor’s prescriptions on treatment of rheumatoid arthritis. *Evidence-Based Complementary and Alternative Medicine*.

[6] Ching H. H., Chun M. L., Tung T. C. (2005). Efficacy and safety of modified mai-men-dong-tang for treatment of allergic asthma. *Pediatric Allergy and Immunology*.

[7] Lv X. H., Wu T., Qin D. Y. (2009). Effect of glycyrrhizin on nitric oxide and nitric oxide synthase in mice model of pronchial asthma. *Lishizhen Medicine and Materia Medica Research*.

[8] Lv X. H., Wu T., Qin D. Y. (2007). The effect of glycyrrhizin on airway inflammation and PLA2 activity in mice asthmatic model. *Lishizhen Medicine and Materia Medica Research*.

[9] Zhang W. Y., Gu Y. C., Tang Y. (2018). Effects of glycyrrhizic acid on ERK1/2 and p38 MAPK signaling pathway in a murine model of asthma. *National Medical Journal of China*.

[10] Wu Q. Z., Tang Y., Zhang J. F. (2014). Therapeutic effects of glycyrrhizic acid on asthma airway inflammation in mice and its mechanism. *National Medical Journal of China*.

[11] Lv X. H., Wu T., Qin D. Y. (2006). Effect of liquorice on airway inflammation and TH1/TH2 imbalance in mouse model. *Chinese Journal of Clinical Pharmacology and Therapeutics*.

[12] Chen W., Ma L., Yang L. S. (2016). Effects of glycyrrhetinic acid on oxidative stress and NF-*κ*B signal path-way in bronchial asthma rats. *Journal of Zhengzhou University (Medical Sciences)*.

[13] Lin H. X., Feng K. Q., Zheng S. G. (2016). Effects of glycyrrhetinic acid on white blood cell count of alveolar lavage fluid and serum related inflammatory factors in asthmatic rats. *Chinese Journal of Gerontology*.

[14] Chen W., Ma L., Yang L. S. (2016). Effects of glycyrrhetinic acid on airway remodeling, Caspase3, Bax and Bcl-2 of lung tissue in bronchial asthma rats. *Pharmacology and Clinics of Chinese Materia Medica*.

[15] Liu B., Wen Q. S., Zhang R. J. (2007). Antiasthmatic effect of isoliquiritigenin and its mechanism. *Chinese Journal of Clinical Pharmacy*.

[16] Huang W.-C., Liu C.-Y., Shen S.-C. (2019). Protective effects of licochalcone A improve airway hyper-responsiveness and oxidative stress in a mouse model of asthma. *Cells*.

[17] Fouladi S., Masjedi M., Ganjalikhani Hakemi M., Eskandari N. (2019). The review of in vitro and in vivo studies over the glycyrrhizic acid as natural remedy option for treatment of allergic asthma. *Iranian Journal of Allergy, Asthma, and Immunology*.

[18] Hosseinzadeh H., Nassiri-Asl M. (2015). Pharmacological effects of Glycyrrhiza spp. and its bioactive constituents: update and review. *Phytotherapy Research*.

[19] Stewart A. G. (2001). Airway wall remodelling and hyperresponsiveness: modelling remodelling in vitro and in vivo. *Pulmonary Pharmacology & Therapeutics*.

[20] Liu C., Weir D., Busse P. (2015). The flavonoid 7,4′-dihydroxyflavone inhibits MUC5AC gene expression, production, and secretion via regulation of NF-*κ*B, STAT6, and HDAC2. *Phytotherapy Research*.

[21] Yang N., Patil S., Zhuge J. (2013 Sep). Glycyrrhiza uralensisFlavonoids present in anti-asthma formula, ASHMITM, inhibit memory Th2 responsesin vitroandin vivo. *Phytotherapy Research*.

[22] Liu C., Yang N., Liang B., Wang R., Song Y., Li X. (2011). Constituents isolated from Glycyrrhiza uralensis inhibit IL-4 and Eotaxin secretion in vitro. *Journal of Allergy and Clinical Immunology*.

[23] Jayaprakasam B., Doddaga S., Wang R., Holmes D., Goldfarb J., Li X.-M. (2009). Licorice flavonoids inhibit eotaxin-1 secretion by human fetal lung fibroblastsin vitro. *Journal of Agricultural and Food Chemistry*.

[24] Yue G. G. L., Chan B. C. L., Kwok H.-F. (2012). Screening for anti-inflammatory and bronchorelaxant activities of 12 commonly used Chinese herbal medicines. *Phytotherapy Research*.

[25] Wei H., Xu D., Yao D. F. (2016). Effect of amygdalin on airway inflammation in mice with allergic asthma. *Shaanxi Journal of Traditional Chinese Medicine*.

[26] Shan L. S. (2019). Amygdalin inhibits inflammation in mice with allergic asthma by regulating MAPK signalling pathway [A]. Chinese association of integrative medicine. Compilation of the 23rd national pediatric academic conference on integrated traditional Chinese and western medicine [C]. *Chinese Association of Integrative Medicine*.

[27] Huang C., Peng W., Wei D. N. (2019). Effect of pinelliae rhizoma praeparatum cum alumine polysaccharides on MUC5AC mRNA in lung tissues of allergic asthma model rats. *Chinese Journal of Experimental Traditional Medical Formulae*.

[28] Chen H., Cheng Y., Yang L. Y. (2018). Experimental study of Asarum on small airway remodeling in asthmatic Guinea pigs. *Journal of Sichuan Traditional Chinese Medicine*.

[29] Chang H.-C., Gong C.-C., Chan C.-L., Mak O.-T. (2013). A nebulized complex traditional Chinese medicine inhibits Histamine and IL-4 production by ovalbumin in Guinea pigs and can stabilize mast cells in vitro. *BMC Complementary and Alternative Medicine*.

[30] Tang Q. F., Liu S. H., Xu X. P., Song S. W. (2013). Effect of earthworm on airway remodeling in the murine model of chronic allergen-induced asthma. *Journal of Guangdong Pharmaceutical University*.

[31] Lu W. W., Wu G. L., Yu G. Y. (2017). Preliminary study on the mechanism of Chinese medicine in treating bronchial asthma. *Zhejiang Journal of Traditional Chinese Medicine*.

[32] Li H. C., Xu R. ’E., Yang Y. N., Wang L. (2010). Effects of earthworm injection on expression of TGF-*β*1/smad2 in bronchial epithelial cells allergized by house dust mite allergen dermatophagoides pteronyssinus antigens. *Chinese Pharmaceutical Journal*.

[33] Wang L., Liu Y., Wang F. (2009). Inhibitory effect of ground dragon on the expression of *α*SMA and FN in the lung tissue of mouse with asthma. *Chinese Journal of Pathophysiology*.

[34] Zhou M. M., Chu X. P., Yang H. Z. (2008). Anti-inflammatory and anti-allergic effects of acidic fraction of Pheratima extract in asthma mice induced by ovalbumin. *China Journal of Chinese Materia Medica*.

[35] Shi Q., Wang X., Liu J. (2019). Anti-asthma effect of an active components group from decoction of Pheretima aspergillum and its chemical composition characterized by liquid chromatography-quadrupole time of flight mass spectrometry. *Iran Journal of Pharmaceutical Research*.

[36] Huang C., Li W., Zhang Q. (2018). Anti-inflammatory activities of Guang-Pheretima extract in lipopolysaccharide-stimulated RAW 264.7 murine macrophages. *BMC Complementary and Alternative Medicine*.

[37] Huang C.-q., Li W., Wu B. (2016). Pheretima aspergillum decoction suppresses inflammation and relieves asthma in a mouse model of bronchial asthma by NF-*κ*B inhibition. *Journal of Ethnopharmacology*.

[38] Su X. D., Jang H.-J., Li H. X., Kim Y. H., Yang S. Y. (2019). Identification of potential inflammatory inhibitors from Aster tataricus. *Bioorganic Chemistry*.

[39] Chen L. S., Zheng D. S. (2015). Bioactive constituents from the rhizomes of aster tataricus L. F. Afford the treatment of asthma through activation of beta(2)AR and inhibition of NFkappa b. *Latin American Journal of Pharmacy*.

[40] Wang Y., Feng D. P., Sun L. B. (2019). Effect of fritillariae cirrhosae bulbus on Notch2 and inflammatory response in asthmatic mice. *Progress of Anatomical Sciences*.

[41] Zhang Y. F., Xu H. N., Huang W. (2018). Effects of fritillariae cirrhosae bulbus on airway inflammation and ERK/MAPK signal pathway in asthma model mice. *China Pharmacy*.

[42] Li H. Z., Gao Z. Y., Huang W. (2017). Effects of fritillariae cirrhosae bulbus on MMP-2, MMP-9 and TIMP-1 in murine asthma model. *China Journal of Chinese Materia Medica*.

[43] Yeum H.-S., Lee Y.-C., Kim S.-H., Roh S.-S., Lee J.-C., Seo Y.-B. (2007). Fritillaria cirrhosa, Anemarrhena asphodeloides, Lee-Mo-Tang and cyclosporine a inhibit ovalbumin-induced eosinophil accumulation and Th2-mediated bronchial hyperresponsiveness in a murine model of asthma. *Basic & Clinical Pharmacology & Toxicology*.

[44] Kim S.-B., Seo Y.-S., Kim H. S. (2019). Anti-asthmatic effects of lepidii seu Descurainiae Semen plant species in ovalbumin-induced asthmatic mice. *Journal of Ethnopharmacology*.

[45] Cai Z. Q., Dai Y., Yuan H. Y. (2006). Experimental study on pharmacodynamics of volatile oil of chenpi. *China Pharmaceuticals*.

[46] Fu M., Zou B., An K. (2019). Anti-asthmatic activity of alkaloid compounds from Pericarpium citri reticulatae (citrus reticulata’Chachi’). *Food & Function*.

[47] Shi Q., Liu Z., Yang Y. (2009). Identification of anti-asthmatic compounds in Pericarpium citri reticulatae and evaluation of their synergistic effects. *Acta Pharmacologica Sinica*.

[48] Duo Z. Y., Wang A. J., Li Q. (2015). Effect of crude and honeyed mori cortex on serum NO, LPO, IL-4 and IFN-*γ* in asthmatic rats. *Chinese Journal of Experimental Traditional Medical Formulae*.

[49] Qin X. Z., Li L. C., Yan G. H., Li G. Z. (2011). Effects of water extract of Cortex Mori to inflammatory cells of bronchoalveolar lavage fluid on model of asthma in mice. *Journal of Medical Science Yanbian University*.

[50] Wei Y. Y., Xu F., Chen X. W. (2009). Antiasthmatic effects of the flavonoid of cortex mori. *Lishizhen Medicine and Materia Medica Research*.

[51] Ma F., Liao D. J., Lei Y. (2018). Effect and mechanism of PDE-4 inhibitor moracin M extracted from cortex mori radicis in mice with asthma. *International Journal of Respiration*.

[52] Kim H.-J., Lee H. J., Jeong S.-J., Lee H.-J., Kim S.-H., Park E.-J. (2011). Cortex Mori Radicis extract exerts antiasthmatic effects via enhancement of CD4+CD25+Foxp3+ regulatory T cells and inhibition of Th2 cytokines in a mouse asthma model. *Journal of Ethnopharmacology*.

[53] Huang L., Wang Y. N., Wu S. Y. (2018). Research progress of pharmacological effects of traditional Chinese medicines ephedrae. *China & Foreign Medical Treatment*.

[54] Li H. Y. (2008). *Effects of Ephedrine Isomers and Cholic Acid Analogs, Used Alone or in Combination, against Histamine Induced Constriction of Guinea Pig Tracheal in Vitro*.

[55] Li Z. Y., Deng J., Xiong B. (2016). Effect of ephedrine on expression of eotaxin in human bronchial epithelial cells stimulated by tumor necrosis factor-*α*. *Chongqing Medicine*.

[56] Xu J. H., Cao H. R., Chen Y. X. (2014). Effect of herba ephedrae or honey-fried herba ephedrae alone on airway inflammation of asthmatic rats. *Journal of New Chinese Medicine*.

[57] Mao Q.-Q., Xu X.-Y., Cao S.-Y. (2019). Bioactive compounds and bioactivities of ginger (zingiber officinale roscoe). *Foods*.

[58] Duan Y. H. (2019). *The Effects and Mechanism of Total Sesquiterpenes from Tussilago Farfara L. on Ova-Sensitized Asthma Model in Rats*.

[59] Choi B.-S., Kim Y.-j., Yoon Y. P., Lee H. J., Lee C. J. (2018). Tussilagone suppressed the production and gene expression of MUC5AC mucin via regulating nuclear factor-kappa B signaling pathway in airway epithelial cells. *The Korean Journal of Physiology & Pharmacology*.

[60] Kim Y., Yeo M., Oh B. (2017). Tussilagone inhibits the inflammatory response and improves survival in CLP-Induced septic mice. *International Journal of Molecular Sciences*.

[61] Li J., Gao W., Gao J. (2017). Metabolomics reveal the protective effect of Farfarae Flos against asthma using an OVA-induced rat model. *RSC Advances*.

[62] Wang L., Du W. N., Guo S. T. (2018). Effects of Platycodon grandiflorum on bronchial remodeling in asthmatic rats. *Chinese Journal of School Doctor*.

[63] Yu W. Y., Zhu H. J. (2012). Study on the pharmacological mechanism of Platycodon grandiflorum to treat bronchial asthma. *Acta Chinese Medicine and Pharmacology*.

[64] Lee H.-Y., Lee G.-H., Kim H.-K., Chae H.-J. (2019). Platycodi radix and its active compounds ameliorate against house dust mite-induced allergic airway inflammation and ER stress and ROS by enhancing anti-oxidation. *Food and Chemical Toxicology*.

[65] Xie Y., Pan H., Sun H., Li D. (2008). A promising balanced Th1 and Th2 directing immunological adjuvant, saponins from the root of Platycodon grandiflorum. *Vaccine*.

[66] Zhang Y. H., Wang J., Ruan H. L. (2007). Effect of verticinone and its derivatives on cyclic adenosine monophosphate of bronchus smooth muscle of Guinea-pig. *Chinese Archives of Traditional Chinese Medicine*.

[67] Lin Y., Wang X. e., Yang X. (2019). The complete chloroplast genome and phylogenetic analysis of Fritillaria ussuriensis (Liliaceae: Fritillaria). *Mitochondrial DNA Part B*.

[68] Chen X., Huang Y. (2013). Effects of Paeonia lactiflora extract on IL-22 and IL-13 levels in asthmatic rat models. *Journal of Military Surgeon in Southwest China*.

[69] Xin Q., Yuan R., Shi W., Zhu Z., Wang Y., Cong W. (2019). A review for the anti-inflammatory effects of paeoniflorin in inflammatory disorders. *Life Sciences*.

[70] Zhou H., Wu Q., Wei L., Peng S. (2018). Paeoniflorin inhibits PDGF-BB-induced human airway smooth muscle cell growth and migration. *Molecular Medicine Reports*.

[71] Ngan L. T. M., Jang M. J., Kwon M. J., Ahn Y. J. (2015). Antiviral activity and possible mechanism of action of constituents identified in Paeonia lactiflora root toward human rhinoviruses. *PLoS One*.

[72] Qin C., Dai X., Yang X. Q. (2020). Intervention effect of honokiol on inflammatory response in lung tissue of asthma mice and its mechanism. *Journal of Jilin University (Medicine Edition)*.

[73] Liu N., Li N., Zhu L. (2019). Effect of honokiol on PI3K/Akt signaling pathway in asthmatic mice and its effect on TLR2 and TLR4 expression. *Chinese Traditional and Herbal Drugs*.

[74] Shen J. L., Man K. M., Huang P. H. (2010). Honokiol and magnolol as multifunctional antioxidative molecules for dermatologic disorders. *Molecules (Basel, Switzerland)*.

[75] Luo H. Y., Wu H. W., Yu X. K. (2019). A review of the phytochemistry and pharmacological activities of Magnoliae officinalis cortex. *Journal of Ethnopharmacology*.

[76] Wu C. L., Wang H. Y., Xu J., Huang J., Chen X., Liu G. T. (2014). Magnolol inhibits tumor necrosis factor-*α*-induced ICAM-1 expression via suppressing NF-*κ*B and MAPK signaling pathways in human lung epithelial cells. *Inflammation*.

[77] Li C., Li C.-J., Ma J. (2019). Magmenthanes A-H: eight new meroterpenoids from the bark of Magnolia officinalis var. Biloba. *Bioorganic Chemistry*.

[78] Ou E. X. (2016). Effect of saikosaponin-d in treatment of cough variant asthma: an experimental study. *Hunan Journal of Traditional Chinese Medicine*.

[79] Bui T. T., Piao C. H., Song C. H., Shin H. S., Chai O. H. (2017). Bupleurum chinense extract ameliorates an OVA-induced murine allergic asthma through the reduction of the Th2 and Th17 cytokines production by inactivation of NF*κ*B pathway. *Biomedicine & Pharmacotherapy*.

[80] Han C., Yang L., Zhang Q. L. (2019). Correlation study on baicalin’s anti-asthmatic effects and HMGB1/TLR4 signal pathway. *World Chinese Medicine*.

[81] Han C., Yang L., Zhang Q. L. (2017). Effect of baicalin on airway remodeling of bronchial asthma. *Chinese Journal of Clinical Pharmacology and Therapeutics*.

[82] Han C., Yang L., Zhang Q. L. (2016). Influence of Baicalin on P38 MAPK signal pathways of rat asthma model. *Guangzhou Medical Journal*.

[83] Wang P. (2016). *The Effect of Baicalin on the Imbalance of Th17/Treg Response in Mice with Allergic Asthma*.

[84] Huang F., Tong X. Y., Deng H. M. (2009). Primary study on mechanism of baicalin on the Th1/Th2 response in murine model of asthma. *Journal of Chinese Medicinal Materials*.

[85] Xing R., Guo F., Tian J. X. (2003). Study on the effect of Scutellaria baicalensis on the pathogenesis of bronchial asthma in rats. *Study Journal of Traditional Chinese Medicine*.

[86] Liu F., Xuan N.-X., Ying S.-M., Li W., Chen Z.-H., Shen H.-H. (2016). Herbal medicines for asthmatic inflammation: from basic researches to clinical applications. *Mediators of Inflammation*.

[87] Ding J. S., Li J. H., Liu X. L. (2007). Preventive effect of Chinonin on Guinea pig’s asthma and influence on ET and NO level. *Chinese Journal of Traditional Medical Science and Technology*.

[88] Lee Y.-C., Kim S.-H., Seo Y.-B., Roh S.-S., Lee J.-C. (2006). Inhibitory effects of Actinidia polygama extract and cyclosporine A on OVA-induced eosinophilia and bronchial hyperresponsiveness in a murine model of asthma. *International Immunopharmacology*.

[89] Wei Y., Tang H. Q., Li X. H., Zhu X. Y. (2013). Effects of loquat leaf injection on T lymphocyte subsets in asthma model mice. *Journal of Emergency in Traditional Chinese Medicine*.

[90] Wu Z. Q. (2015). *Study on the Anti-allergic Effect and Mechanism of Different Extraction from Ramulus Cinnamomi*.

[91] Lv X., Xu G. Y., Wang C. M. (2020). Main targets and active components of schisandra chinensis for asthma treatment based on network pharmacology. *Journal of Beihua University (Natural Science)*.

[92] Wang D. R., Wang Y. T., Hua S., Fan X. Y. (2019). Effect of Schisandrin B on lung inflammation in asthmatic mice and its mechanism. *Acta Universitatis Medicinalis Anhui*.

[93] Chen X., Feng J. (2013). Effects of Schisandra chinensis extract on blood antioxidant activity of asthmatic model rats. *China Pharmaceuticals*.

[94] Ahmed H. (2019). Ethnomedicinal, phytochemical and pharmacological investigations of Perilla frutescens (L.) britt. *Molecules*.

[95] Global Initiative for Asthma, Available from (2006). *Global Strategy for Asthma Management and Prevention*.

[96] Zhang J. A., Li J. Y., An X. D. (1997). Analysis of the famous prescriptions of asthma in ancient and modern Chinese and Chinese. *Chinese Journal of Information on TCM*.

[97] Agbabiaka T. B., Spencer N. H., Khanom S., Goodman C. (2018). Prevalence of drug-herb and drug-supplement interactions in older adults: a cross-sectional survey. *British Journal of General Practice*.

[98] Lim J., Chee S., Wong W., He Q., Lau T. (2018). Traditional Chinese medicine: herb-drug interactions with aspirin. *Singapore Medical Journal*.

[99] Chua Y., Ang X., Zhong X. M. (2015). Interaction between warfarin and Chinese herbal medicines. *Singapore Medical Journal*.

[100] Cheng F., Wang X., Song W. (2014). Biologic basis of TCM syndromes and the standardization of syndrome classification. *Journal of Traditional Chinese Medical Sciences*.

[101] Jiang L., Liu B., Xie Q. (2013). Investigation into the influence of physician for treatment based on syndrome differentiation. *Evidence Based Complementary and Alternative Medicine*.

[102] Borish L. (2016). The immunology of asthma. *Annals of Allergy, Asthma & Immunology*.

[103] A.V R. (2014). Inhalational steroids and iatrogenic cushing’s syndrome. *The Open Respiratory Medicine Journal*.

[104] Yeh Y.-H., Chou Y.-J., Huang N., Pu C., Chou P. (2016). The trends of utilization in traditional Chinese medicine in Taiwan from 2000 to 2010. *Medicine*.

